# Evaluation of the surface roughness and dimensional accuracy of low-cost 3D-printed parts made of PLA–aluminum

**DOI:** 10.1016/j.heliyon.2024.e25508

**Published:** 2024-02-05

**Authors:** Nor Aiman Sukindar, Ahmad Shah Hizam Md Yasir, Muhammad Danial Azhar, Muhammad Afif Md Azhar, Nor Farah Huda Abd Halim, Mohd Hafis Sulaiman, Ahmad Syamaizar Haji Ahmad Sabli, Mohd Khairol Anuar Mohd Ariffin

**Affiliations:** a School of Design Universiti Teknologi Brunei Jalan Tungku Link Gadong, BE1410, Brunei Darussalam; b Faculty of Resilience Rabdan Academy, 65, Al Inshirah, Al Sa’adah, Abu Dhabi, 22401, PO Box: 114646, Abu Dhabi, United Arab Emirates; c Department of Manufacturing and Materials Engineering, International Islamic University Malaysia, 53100, Jalan Gombak, Kuala Lumpur, Malaysia; d Department of Mechanical and Manufacturing Engineering, Faculty of Engineering. Universiti Putra Malaysia, 43400 UPM Serdang, Selangor, Malaysia

**Keywords:** Surface roughness, Fused deposition modeling, Aluminum composites

## Abstract

Fused deposition modeling (FDM) is currently used in several fields, such as architecture, manufacturing, and medical applications. FDM was initially developed to produce and create prototypes, but the expense appears excessive for producing final products. Nevertheless, in this day and age, engineers have developed a low-cost 3D printer. One of the major issues with low-cost 3D printers is the low dimensional accuracy and high tolerances of the printed products. Herein, different printing parameters, i.e., layer thickness, printing speed, and raster angle, need to be investigated to enhance the surface roughness of the parts produced using FDM. Thus, the present study focuses on investigating the performance of the surface finish produced by FDM by manipulating different parameters such as layer thickness, printing speed, and raster angle. Taguchi's method, based on the L_9_ array for experimental design, was employed to elucidate the response variables. The sample model was developed following ISO standards, utilizing polylactic acid (PLA)–aluminum as the filament material. The analysis of variance results indicated that the layer thickness and raster angle significantly affect the surface roughness of the printed parts, with statistical *P*-values of 0.016 and 0.039, respectively. This enables an easy selection of the optimal printing parameters to achieve the desired surface roughness. The dimensional accuracy of the fabricated part was also evaluated. Thirteen dimensions of the part features were analyzed, and the results showed that the FDM machine exhibited good accuracy for most of the shapes, with a deviation below 5%.

## Highlights

• Surface roughness in PLA–aluminum is mainly affected by layer thickness and raster angle.
• FDM machine has low accuracy for producing circular shapes like cylinders and holes.
• Lower layer thickness and raster angle have the most significant impact on the accuracy of the printed PLA-aluminum part.

## Graphical abstract

**Figure:**
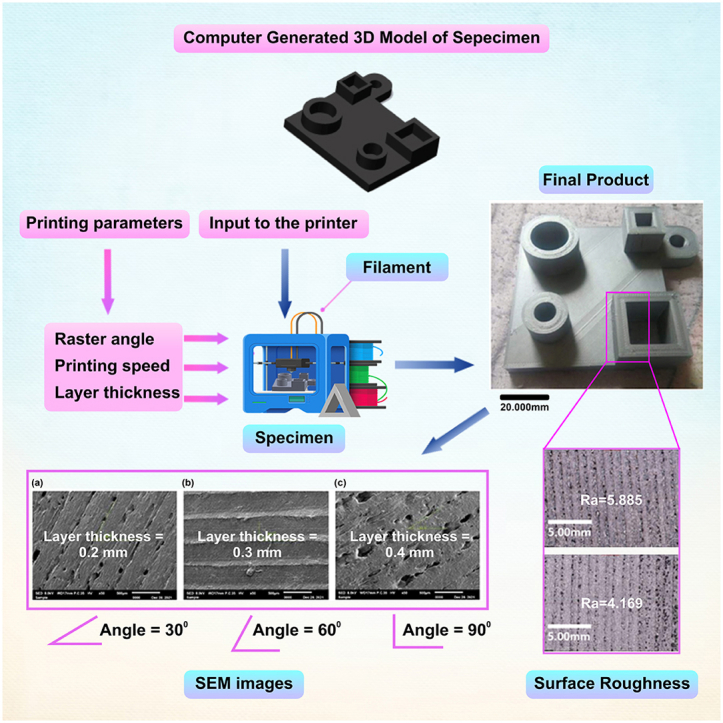

## 1 Introduction

Three-dimensional (3D) printing, also known as additive manufacturing, was introduced in the 1980s [1]. The demand for 3D-printed parts significantly increased over the years. Several industries utilize 3D printing to create prototypes for their products, benefiting from advantages like reduced production costs and time. Generally, AM technology is categorized into three primary types: powder-based, liquid-based, and solid-based. This technology comprises methods such as material extrusion (also known as fused deposition modeling - FDM), stereolithography, selective laser melting, and selective laser sintering [2]. Stereolithography (SLA) was invented by Hideo Kodama [3], selective laser melting (SLM) was introduced by the Fraunhofer Institute ILT [4], laminated object manufacturing was introduced by Helisys Inc. [5], and fused deposition modelling (FDM) was invented by Scott Crump [6].

Stereolithography, selective laser melting, and selective laser sintering are three additive manufacturing methods that have revolutionized the creation of 3D items. Stereolithography, also called SLA, creates 3D objects by preferential solidifying liquid resin using photopolymerization approaches [7]. Huang and colleagues additionally noted that this method can produce items with excellent dimensional precision and accuracy, even with different materials. This technology has been used in various applications, including the field of medicine and the production of small-scale pH sensors [8]. Meanwhile, selective laser melting (SLM) relies on a high-energy laser to fully liquefy and merge metal powders for fabricating intricate metal components with superior mechanical characteristics [9].

Besides, selective laser sintering (SLS) selectively fuses powdered material, such as plastic and metal, using a high-power laser to produce a 3D structure. The methods have been utilized to print different items across several applications in the medical sector, including the formation of pharmaceutical dosage [10]. The progressive improvements in additives fabrication approaches have now led to better production of 3D objects. For example, using high-speed 3D printing processes, properties like sub-voxel-scale precision and deep subwavelength surface roughness can be acquired through the projection micro-stereolithography process and grayscale photopolymerization [11].

Among the available 3D printers, FDM stands out as one of the most popular choices among users. FDM devices are known as “desktop 3D printers” because they are inexpensive compared to other 3D printing methods, and consequently, they are commonly used in the industry. The materials used in FDM are cheaper than those used in melting and sintering technologies [[12], [13], [14]]. Thus, FDM is the most cost-effective technology for prototype production. The FDM lead times are short because of the high availability of the technology [15]. The wide range of materials available for FDM (ranging from commodity thermoplastics, such as acrylonitrile butadiene styrene (ABS) and polylactic acid (PLA), to engineering materials, such as polyethylene terephthalate glycol (PETG) and polyamide (PA), and high performance materials, such as polyetherimide (PEI) and polyetheretherketone (PEEK)) is a main advantage of this technology [[16], [17], [18], [19], [20]]. Other materials such as metal composites, are also suitable for FDM. Metal–polymer composite materials are suitable for FDM-based manufacturing because they have both polymeric and metallic properties. The intermediate properties of metal–polymer composites can be useful in applications where no other class of materials is suitable because of cost or time constraints. Additional benefits of these technologies include design flexibility, mass customization, low cost, and short commercialization time. A printed product based on a metal–polymer composite is mechanically strong and rigid, and it exhibits superior mechanical properties to PLA- or ABS-based products, resulting in a longer useable life and better dimensional stability. Filament materials based on metal–polymer composites have recently emerged. These filament materials include aluminum–PLA, iron–ABS, iron–nylon, and aluminum–epoxy [[21], [22], [23], [24]].

Currently, several open-source design software is used for numerous products and initiatives. This initiative started with the creative innovation by the RepRap community. After the expiration of the FDM patents, the open-source 3D printer era began. A new inexpensive technology called replicating rapid prototyper (RepRap) was created and was sold for the first time in November 2008 [25]. The first and second RepRap versions were “Darwin” and “Mendel,” respectively. This marks the onset of low-cost FDM printers, and their demands from users either for personal use or small business have increased dramatically over the years. However, these low-cost FDM printers possess some drawbacks, opening up the opportunity to study the performance of low-cost FDM printers from various aspects, especially in terms of dimensional accuracy and surface roughness [26]. Numerous variables can affect the dimensional accuracy of parts manufactured using FDM. The layer thickness and orientation, support structure, and extrusion angle are usually investigated to test the dimensional accuracy of the final printed products [27]. One of the studies has investigated the accuracy of FDM-printed parts using PLA by manipulating several parameters, including layer thickness and printing temperature [28]. The findings show that high printing temperature reduces the accuracy of the printed parts due to the material's higher degree of melting compared to that at the lower printing temperature. Other studies have investigated the effect of printing parameters, including layer thickness, nozzle diameter, gap between layers, and extrusion angle, on the FDM, and their findings show that small layer thicknesses result in a better surface quality [29]. A similar study was conducted using different polymers to analyze the dimensional accuracy and surface roughness of the FDM-printed parts. Polymers used for the study include PLA, PLA+, ABS, and ABS+, and the results show that PLA + shows better accuracy for the parts, while ABS exhibits high surface roughness [30]. All these studies have focused on the polymer alone, but the industry is investigating various materials to suit different applications.

Engineering and industrial demands for composite materials are very high because of their light weight as well as high strength, hardness, and wear resistance. Among composites, aluminum matrix composites are highly required in the engineering field. Because of their excellent tribological, mechanical, and physical properties [31,32]. Various polymer composites have been developed recently such as metal-filled PLA for different applications and usage. Several studies have been conducted with PLA metal composites to investigate the mechanical performance and dimensional accuracy of printed parts using a low-cost 3D printer. Research has been performed to investigate the quality of the surface texture of PLA–copper by manipulating several printing parameters, including printing speed, printing temperature, bed temperature, layer thickness, and raster angle [33]. The finding shows that the raster angle, layer thickness, and bed temperature can considerably affect the surface roughness, and to obtain an optimal surface roughness for printing the PLA–copper, they should be set at 90°, 0.1 mm, and 90 °C, respectively. However, this study did not analyze the performance of the dimensional accuracy of the printed parts. Another study has analyzed the mechanical performance of the printed parts using PLA–aluminum [34]. This study manipulated several printing parameters, and its results show that the number of the shell has the greatest effect on the tensile strength. The printed parts of PLA–aluminum have been compared with the normal PLA, and the results show that the tensile strength of normal PLA is 20% higher than the PLA–aluminum. This is due to the inconsistent layers of printed parts, leading to less rigid structure. Hence, the above studies show that different studies need to be performed to analyze the overall performance of these materials from different aspects, especially in terms of dimensional accuracy and surface roughness, to obtain the maximum benefits of PLA–aluminum. Currently, 3D printing is transitioning into rapid production, increasing the demand for high-quality surface roughness of printed parts and making studies in this area essential. Surface roughness is a major factor that helps customers to determine the quality of the final product [35].

The surface roughness and dimensional accuracy of FDM should be further improved. Prototype parts manufactured using FDM have a very high surface roughness compared to those manufactured by other processes. Moreover, some inaccuracies often occur during the printing process, which results in surface warping and inaccurate dimensions as reported in a previous study [26]. Several recent studies have investigated the printing parameters that affect the surface roughness of PLA-printed parts using the FDM method for dental implants. The findings show that the higher printing temperature and lower layer thickness resulted in lower surface roughness [36]. It was suggested that a printing temperature of 230 °C and a layer thickness of 0.16 mm improved the surface roughness. A similar study reported that layer height and flow rate affect the surface roughness [37]. The study suggested that lower layer thickness is better for surface roughness for vertically inclined surfaces, and a 100% flow rate provides an optimized flat roughness value. Additionally, similar research findings indicate that a lower layer thickness contributes to a reduced roughness value and suggest that a low flow rate can help minimize dimensional errors [38].

Based on the literature study above, optimizing the printing parameter is crucial for achieving the desired quality of printed parts. Each material needs different printing parameters, leading to sustainability issues if not optimized. One of the issues is the generation of waste due to multiple trials and errors, leading to increased energy consumption. A recent study shows the importance of optimizing the FDM printing parameters concerning sustainability issues, such as energy consumption and waste generated [39]. Most studies investigate the printing parameter using low-cost FDM for typical polymers, such as PLA and ABS, while this study focuses on polymer composite. Therefore, this present study focuses on manipulating the printing parameters to enhance the quality of the surface roughness of PLA–aluminum. The study will concentrate on layer thickness, raster angle, and printing speed, utilizing ANOVA to analyze and identify the response variables. The results were then analyzed, and the optimum printing parameters were determined. A dimensional accuracy test will be performed to validate the optimized parameters, and the performance of low-cost FDM will be observed based on the accuracy measurement.

## 2 Materials and methods

### 2.1 Experimental setup

This study is conducted to analyze the surface roughness and dimensional accuracy of parts printed using FDM. An ISO-standard Solidworks file (Solidworks is engineering software) that was converted to an STL file was used as the 3D model and was printed using desktop 3D printers. The desktop 3D printer used in this experiment was Artillery Sidewinder X1 3D printer with a build volume of 300 mm × 300 mm × 400 mm. A default nozzle diameter size of 0.4 mm, provided by the manufacturer, was used for experiments. A bed temperature of 75 °C is kept constant throughout the experiment. The number of contours is set to be 3, the infill pattern is set as a line pattern, and the infill flow rate is 100%. The material used for this experiment was PLA–aluminum (Flashforge, USA) in silver color with 1.75-mm diameter, and the printing temperature was 200 °C. The parts were printed, and the surface roughness (Ra) was measured using Alicona InfiniteFocusSL with a 0.25 mm cut-off length. The surface roughness test was performed to obtain the average Ra value. For dimensional accuracy, the features were examined from the nominal dimension, including the accuracy of the height (mm), width (mm), thickness (mm), and roundness (mm). The results were recorded and analyzed. The vernier calipers used were MITUTOYO 530-123 with 0.02 mm resolution and MITUTOYO 112–255 for micrometers with 0.001 mm resolution.

### 2.2 Sample preparation

**Fig. 1:**
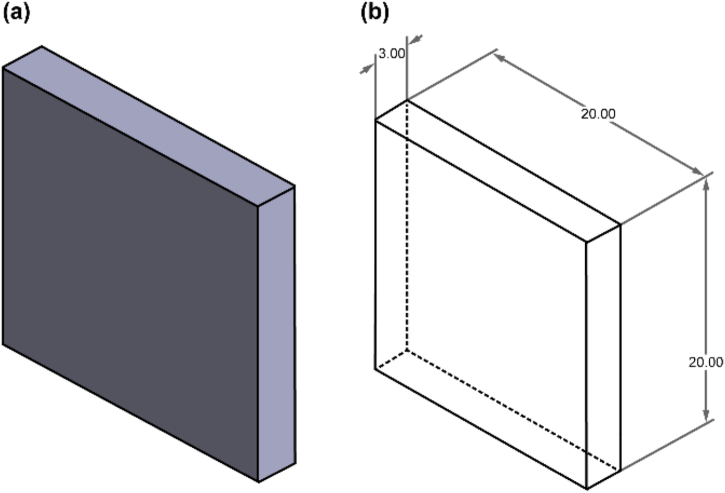
Model sample used in the surface roughness test: (a) 3D model of the test sample, and (b) Dimensions of the test sample.

**Fig. 2:**
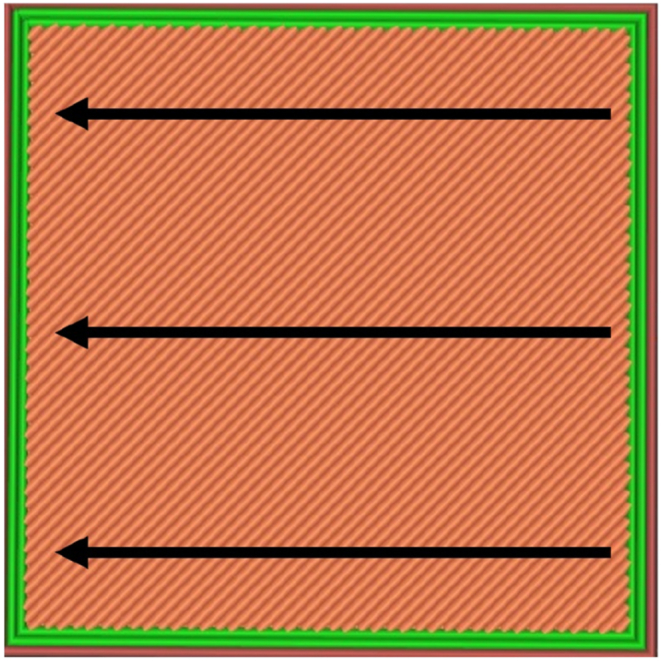
The direction for measuring the surface roughness value for sample parts.

For the surface roughness test, a sample model was generated in Solidworks based on the standard straightness and flatness specifications in ISO 12780 and ISO 12781, respectively. The sample dimensions are 20.0 mm × 3.0 mm × 20.0 mm. As shown in Fig. 1, the circular ISO-standard has not been implemented because the flatness standard provides greater dimensional accuracy compared to the circular one. This study examined several printing parameters, i.e., printing speed, layer thickness, and raster angle. The printing speed was set to 30, 60, and 90 mm/s, whereas the controlled layer thicknesses were 0.2, 0.3, and 0.4 mm, and the modified raster angle values were 30°, 60°, and 90°. The surface roughness value was measured perpendicular to the build direction along three different areas, and the average values were calculated for each sample. Fig. 2 shows the framework for measuring the surface roughness value.

The parameters involved in this study were layer thickness (mm), printing speed (mm/s), and raster angle (degree). The design of experiment was performed using Minitab 18.0 (Minitab, USA) software. Based on the Taguchi's method in 3^3 design factors, the total numbers of samples to be printed were nine. The setup parameters are shown in Table 1.

**Table 1:** Three parameters varied for measuring surface roughness.

Level	Layer Thickness (mm) [A]	Printing Speed (mm/s) [B]	Raster Angle (degrees) [C]
1	0.2	30	30
2	0.3	60	60
3	0.4	90	90

**Fig. 3:**
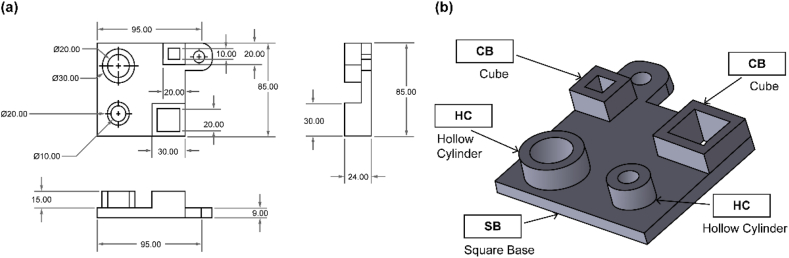
Benchmark model for dimensional accuracy: (a) Technical drawing of the benchmark model, and (b) 3D view of the benchmark model.

For the dimensional accuracy test, another 3D model was created using Solidworks according to ISO standards. Straightness, flatness, roundness, and cylindrical shape were measured according to ISO 12780, ISO 12781, ISO 12181, and ISO 12180, respectively. Each ISO-standard specifies different speeds, sizes, and dimensions. Thus, based on the ISO standards, the specimen part exhibited the following dimensions: 85 mm × 80 mm × 9 mm. As shown in Fig. 3, the proposed design has different features to allow for accurate control of different geometrical qualities and product specifications. The characteristics and ISO standards used for the 3D-printed reference model are listed in Table 2.

**Table 2:** Standard specifications of the benchmark model.

Feature Type	Total	ISO-Standard
Hollow Cylinder (HC)	2	ISO 12181 (Roundness Measurement) ISO 12180 (Cylindrical Shape Measurement)
Cube (CB)	2	ISO 12780 (Straightness Measurement) ISO 12781 (Flatness Measurement)
Square Base (SB)	1	ISO 12780 (Straightness Measurement) ISO 12781 (Flatness Measurement)

## 3 Results

### 3.1 Surface roughness test

The surface roughness, Ra, value of the printed parts was determined through this experiment. Table 3 shows the arithmetic average of all the Ra values obtained at different printing parameters.

**Table 3:** Ra values of the printed samples.

Sample No.	Layer Thickness (mm)	Printing Speed (mm/s)	Raster Angle (°)	Average Ra Value (μm)	Image of the Sample Surface Roughness
1	0.2	30	30	5.885	
2	0.2	60	60	4.169	
3	0.2	90	90	4.342	
4	0.3	30	60	2.726	
5	0.3	60	90	4.175	
6	0.3	90	30	9.426	
7	0.4	30	90	9.153	
8	0.4	60	30	13.488	
9	0.4	90	60	10.341	

The highest and lowest Ra values were 13.488 and 2.726 μm obtained on Run 8 and Run 4, respectively. These results confirm the significant effect of the layer thickness, printing speed, and raster on the Ra values. For further investigation, an analysis of variance (ANOVA) was conducted using Minitab software. Table 4 shows the ANOVA results of the transformed response.

**Table 4:** Analysis of variance of the transformed response.

Source	DF^a^	Adj SS^b^	Adj MS^c^	F-Value	*P*-Value
Layer Thickness (mm)	2	69.617	34.8087	60.61	0.016
Printing Speed (mm/s)	2	6.888	3.4440	6.00	0.143
Raster Angle (°)	2	28.638	14.3192	24.93	0.039
Error	2	1.149	0.5743		
Total	8	106.293			

ANOVA table was prepared to investigate the experimental data to determine the parameters that affect the surface roughness. Based on this analysis, it is clearly shown that the layer thickness and raster angle are the most significant factors affecting Ra value, with a *P*-value of 0.016 and 0.039, respectively. In contrast, the printing speed is considered insignificant as the *P*-value exceeds 0.05.

a Degree of freedom.

b Adjusted sum of squares.

c Adjusted mean squares.

**Fig. 4:**
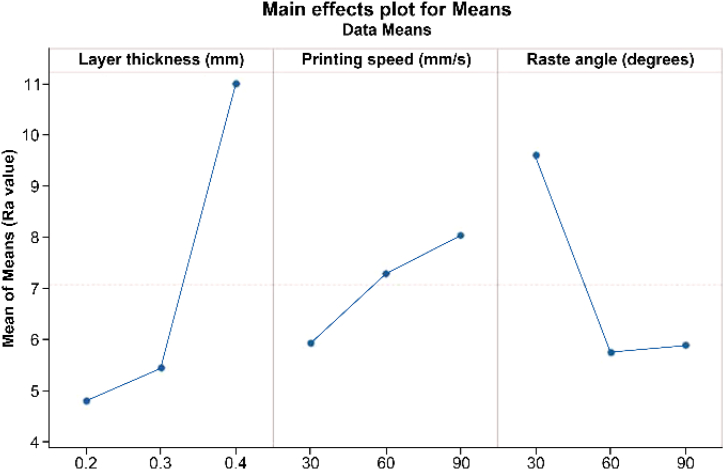
Effect of the main printing parameters on surface roughness.

**Fig. 5:**
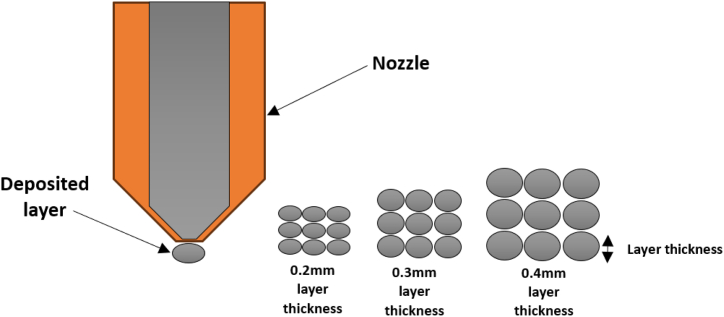
Schematic representation of deposited different layer thickness.

Fig. 4 shows the relationship among the three parameters. The most significant printing parameters affecting the Ra value were determined based on the means of the response value. The lowest Ra value was obtained at a layer thickness of 0.2 mm. The Ra value increased significantly with the increment in layer thickness to 0.4 mm. Layer height will affect the resolution of the printed parts; a lower layer height produces a smoother surface and fine resolution compared to a higher layer height. This result aligns with previous research, which demonstrated that lower layer thickness contributes to lower Ra value [40,41]. Fig. 5 shows the schematic representation of different layer thicknesses deposited using FDM. It is clear that different resolutions ranging from 0.2 mm to 0.4 mm have a noticeable impact on the Ra value.

For the raster angle, the lowest Ra value was obtained at 60°, while the highest was at 30°. The significant difference in Ra value between the 30° and 60° raster angles is primarily due to the measurement of layers nearly parallel to the direction of roughness. This is also reported in the previous study, where similar findings were based on the simulation and experimental value [42]. Raster angles play a major part in determining the quality of the printed parts. Based on the previous study, the 90° raster angle provides the lowest Ra value when measured parallel to the raster angle [43]. This explains why the higher degree of raster angle tends to have the lowest Ra value.

The third parameter examined in this study is printing speed. Although this parameter does not significantly affect the Ra value according to the ANOVA analysis, it is still crucial to discuss its outcome. Higher Ra values were obtained at 90 mm/s, whereas the lowest Ra value was obtained at 30 mm/s as shown in Fig. 4. Printing speed determines the movement of the printed head or extruder when depositing layer by layer. Higher speed provides higher Ra value because the extruded material cannot be deposited properly due to the fast movement of the printer head. The flow of the material inside the extruder will be unstable, creating a rough surface. This finding also conforms to a previous study, which demonstrated that the higher printing speed contributes to a higher surface roughness value [44].

According to the data listed in Table 4, the layer thickness is the most significant factor affecting Ra, followed by the raster angle and printing speed. Based on this analysis, the printing parameters can be tuned to achieve the optimum surface quality of the printed parts. According to the ANOVA analysis, the layer thickness and raster angle are the significant printing parameters. Table 5 shows the ranking factors that affect the surface roughness, as layer thickness is the main factor, followed by raster angle and printing speed.

**Table 5:** Response table for means printing parameters.

Level of Parameter	Layer Thickness (mm)	Printing Speed (mm/s)	Raster Angle (°)
1	4.799	5.921	9600
2	5.442	7.277	5.745
3	10.994	8.036	5.890
Delta	6.195	2.115	3.854
Rank	1	3	2

**Fig. 6:**
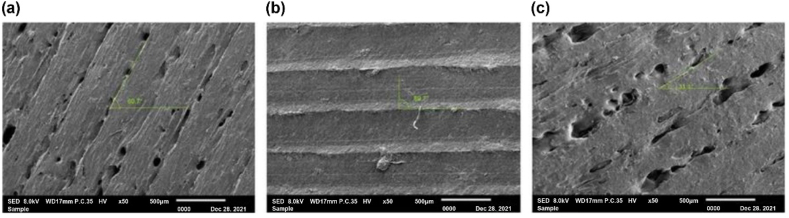
Scanning electron microscopy images of the surface roughness at different raster angles: (a) 30°, layer thickness = 0.2 mm, (b) 60°, layer thickness = 0.3 mm, and (c) 90°, layer thickness = 0.4 mm.

According to the scanning electron microscopy images in Fig. 6, the lowest Ra value was obtained at a layer thickness of 0.2 mm. The figure also shows the changes in Ra with the raster angle (30°, 60°, and 90°).

**Fig. 7:**
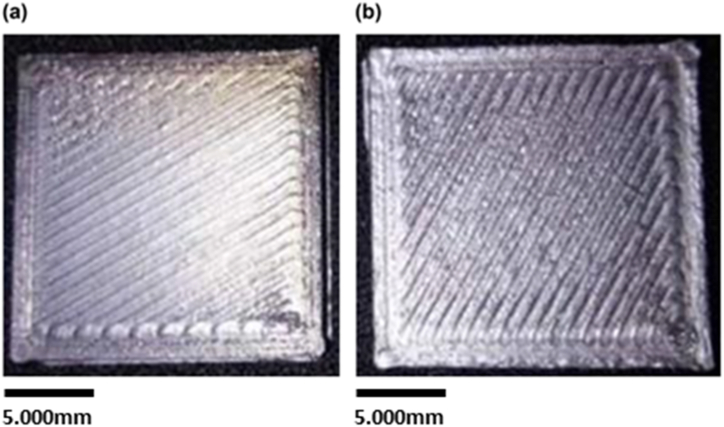
The printed samples with the (a) lowest and (b) highest Ra values.

**Fig. 8:**
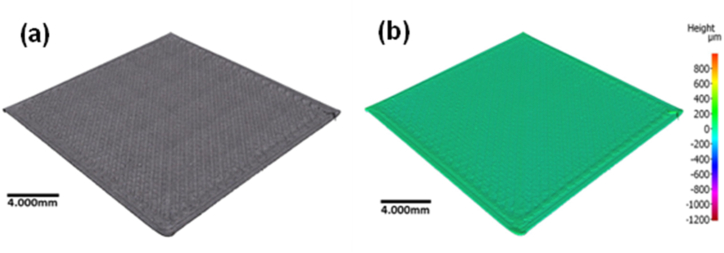
The newly printed sample based on optimum printing parameters: (a) surface of printed parts and (b) Ra value.

Therefore, a fine surface quality can be achieved by setting the layer thickness, raster angle, and printing speed to 0.3 mm, 60°, and 30 mm/s, respectively. This confirms the possibility of controlling the surface roughness of the printed sample by selecting the optimal parameters for 3D printing. Fig. 7 shows a comparison between the surface quality of the printed parts with the lowest and highest Ra values. In addition, the new sample was fabricated based on optimal printing parameters, including a printing speed of 30 mm/s, raster angle of 60°, and layer thickness of 0.2 mm. Its Ra value was compared to the sample that possesses the lowest Ra value; here, the newly fabricated sample exhibits an average Ra value of 1.789 μm compared to the best sample, whose value is 2.726 μm. This shows that tuning printing parameters can result in a smoother surface. Fig. 8 shows the new sample fabricated based on the optimal printing parameters

**Fig. 9:**
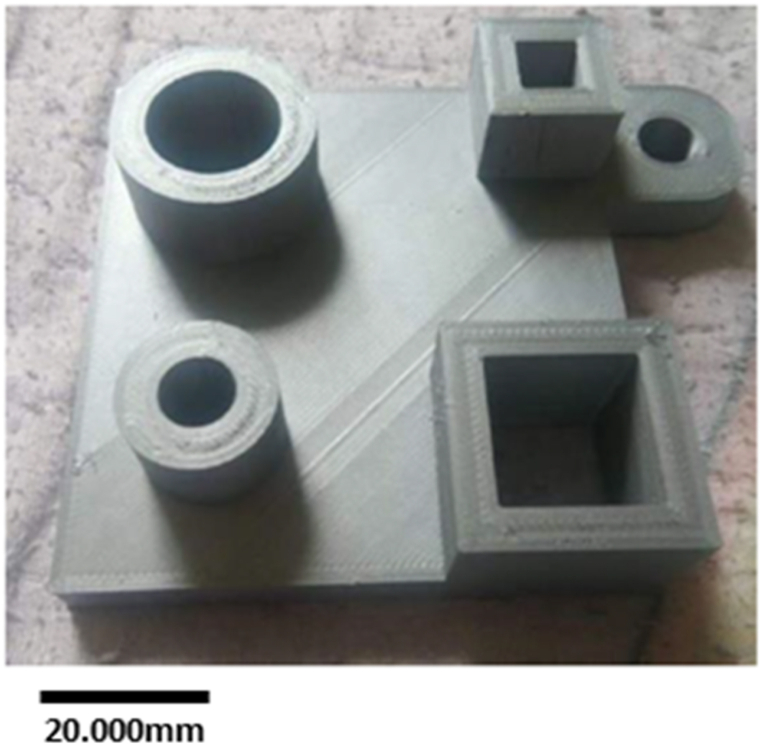
A printed sample made of an aluminum-composite.

### 3.2 Dimensional accuracy test

Fig. 9 shows the sample printed based on the ISO-standard. The dimensional accuracy of the printed sample was evaluated by comparing its dimensions to those specified in the design. For this test, the printing speed is set at 30 mm/s, the layer thickness is 0.2 mm, the printing temperature is 200 °C, the infill density is 20%, and the nozzle diameter is 0.4 mm. These settings are set based on the optimum setting parameters.

The overall dimensional accuracy of Artillery Sidewinder X1 3D printer in terms of height (mm), width (mm), thickness (mm), and roundness is presented in Table 6, Table 7. The difference between the measured height and that specified in the model is less than 0.1 mm. The difference between the measured and designed width and thickness is approximately 0.3 mm. The highest variation between the measured and design roundness is 0.4–0.5 mm.

**Table 6:** Dimensional accuracy of height, width, and thickness of the printed sample.

Feature Type	Design Dimension	Reading 1	Reading 2	Reading 3	Average	Difference	Measuring Tools
Height (mm)							
Hollow Cylinder 1	15	14.968	14.889	14.960	14.939	0.061	Micrometer
Hollow Cylinder 2	15	14.930	14.923	14.930	14.927	0.073	Micrometer
Cube 1	15	15.045	15.039	15.033	15.039	0.039	Micrometer
Cube 2	15	14.944	14.867	14.943	14.918	0.082	Micrometer
Width (mm)							
Cube 1 (Inside)	20	20.010	20.030	20.010	20.016	0.016	Vernier Caliper
Cube 1 (Outside)	30	30.100	30.100	30.090	30.096	0.096	Vernier Caliper
Cube 2 (Inside)	10	9.650	9.550	9.650	9.617	0.383	Vernier Caliper
Cube 2 (Outside)	20	20.040	20.020	20.040	20.033	0.033	Vernier Caliper
Square Base	85	85.490	84.670	85.330	85.163	0.163	Vernier Caliper
Thickness (mm)							
Square Base	9	8.794	8.855	8.862	8.837	0.163	Micrometer

**Table 7:** Roundness accuracy of the printed sample.

Feature Type	Design Dimension	Lower Region	Middle Region	Upper Region	Average	Difference	Measuring Tools
Roundness (mm)							
Hollow Cylinder 1 (Inside)	20	19.560	19.640	19.590	19.597	0.403	Vernier Caliper
Hollow Cylinder 1 (Outside)	30	29.920	29.890	29.880	29.897	0.103	Vernier Caliper
Hollow Cylinder 2 (Inside)	10	9.560	9.380	9.430	9.457	0.543	Vernier Caliper
Hollow Cylinder 2 (Outside)	20	20.020	20.010	20.020	20.017	0.017	Vernier Caliper

The results indicate that the deviation percentage in the height of the printed parts was relatively low (0.26%–0.54%). Higher deviation percentages were observed for width and thickness (0.08%–3.83%). Finally, the roundness measurement exhibited the highest deviation percentage (0.085%–5.43%) for hollow cylinders 2 (inside). This result shows that FDM has few limitations for printing certain sizes and shapes. The deviation value for a hollow cylinder 2 shape (inside) can be seen clearly in Fig. 8, exceeding 5% deviation. Furthermore, this is similar to hollow cylinder 1 (inside), demonstrating a 4% deviation from the nominal dimension. These results are similar to findings from a previous study, which revealed that the roughness of the part increases with decreasing size [45]. This is assumed to be due to the severe influence of the residual stress developed in the nozzle on small parts and is affected by the deposition flow rate [46,47]. The default setting used in the low-cost 3D printer for the flow rate cannot meet all the requirements for different geometries and sizes. Another factor considered to be affecting the part geometry is the road width. A previous study has shown that increasing road width will increase the residual stress and cause distortion to the printed parts [48]. However, this factor is not a controllable factor in this study. Overall, results of the present study show promising performance for the low-cost 3D printer as most of the printed parts and features are within 5% deviation from the nominal value, meeting the performance of different commercial desktop 3D printers used in a previous study [49].

## 4 Conclusions

In this study, the optimal 3D-printing parameters were selected based on the Taguchi method. Three printing parameters were selected: layer thickness, raster angle, and printing speed to observe their impact on the Ra value and dimensional accuracy when printing PLA-aluminum.

The main conclusions of this study are as follows:

• Layer thickness and raster angle significantly affect the Ra value in printing PLA-aluminum.
• Lower layer thickness provides a lower Ra value due to a smooth surface compared to higher layer thickness.
• Ra value is lower when the raster angle is near the parallel to the direction of roughness being measured.
• Low-cost FDM can produce high-quality prints with good tolerance by adjusting the appropriate printing parameters.
• The study shows that the tolerance of printed parts is within 5% deviation, and the differences between the measured and design dimensions of the printed products were relatively small (difference = 0.1–0.5 mm).

Overall, this study aims to facilitate the advancement and global expansion of 3D printers by offering solutions to overcome their limitations. The authors suggest exploring more parameters, such as printing temperature, road width, and others, that can impact the surface roughness and dimensional accuracy of the printed parts.

## Funding

This research received funding from Research Management Centre, 10.13039/501100007297International Islamic University Malaysia (project number RMCG20-033-0033).

## Institutional review board statement

Not applicable.

## Data availability statement

The authors declare that all data are included in the article, and no additional data is available.

## CRediT authorship contribution statement

**Nor Aiman Sukindar:** Validation, Supervision, Software, Project administration, Methodology, Investigation, Conceptualization. **Ahmad Shah Hizam Md Yasir:** Writing – review & editing. **Muhammad Danial Azhar:** Writing – original draft, Software, Methodology, Formal analysis, Data curation. **Muhammad Afif Md Azhar:** Writing – original draft. **Nor Farah Huda Abd Halim:** Visualization, Resources. **Mohd Hafis Sulaiman:** Visualization. **Ahmad Syamaizar Haji Ahmad Sabli:** Funding acquisition. **Mohd Khairol Anuar Mohd Ariffin:** Writing – original draft.

## Declaration of competing interest

The authors declare the following financial interests/personal relationships which may be considered as potential competing interests:NOR AIMAN SUKINDAR reports financial support was provided by 10.13039/501100007297International Islamic University Malaysia. NOR AIMAN SUKINDAR reports a relationship with International Islamic University Malaysia that includes: employment. If there are other authors, they declare that they have no known competing financial interests or personal relationships that could have appeared to influence the work reported in this paper.
